# Assessment of performance and comparison of three commercial HDV RNA detection assays: implications for diagnosis and treatment monitoring

**DOI:** 10.3389/fcimb.2024.1422299

**Published:** 2024-06-26

**Authors:** Marta Illescas-López, Lucía Chaves-Blanco, Adolfo de Salazar, Melisa Hernández-Febles, Raquel Carracedo, Eduardo Lagarejos, Ana Fuentes, Sara Pereira, Maria Cea, Alberto De La Iglesia, Carolina Freyre, Asunción Iborra, Valle Odero, Aurora García-Barrionuevo, Antonio Aguilera, María José Pena, Federico García

**Affiliations:** ^1^ Servicio de Microbiología, Hospital Universitario San Cecilio, Granada, Spain; ^2^ Instituto de Investigación Biosanitaria Ibs.Granada, Granada, Spain; ^3^ Centro de Investigación Biomédica en Red en Enfermedades Infecciosas (CIBERINFEC), Instituto de Salud Carlos III (ISCIII), Madrid, Spain; ^4^ Servicio de Microbiología, Hospital Universitario de Gran Canaria Doctor Negrín, Las Palmas de Gran Canaria, Spain; ^5^ Servicio de Microbiología, Hospital Clínico Universitario de Santiago de Compostela, Santiago de Compostela, Spain; ^6^ Servicio de Microbiología, Hospital Infanta Elena, Huelva, Spain; ^7^ Servicio de Microbiología, Hospital de Puerto Real, Puerto Real, Spain; ^8^ Servicio de Microbiología, Hospital Universitario Virgen de la Arrixaca, Murcia, Spain; ^9^ Servicio de Microbiología, Hospital Universitario de Jerez de la Frontera, Jerez de la Frontera, Spain; ^10^ Servicio de Microbiología, Hospital Universitario Virgen de la, Victoria, Málaga, Spain

**Keywords:** hepatitis delta virus, molecular diagnosis, RT-PCR, viral hepatitis, HDV screening

## Abstract

**Objectives:**

Precise HDV-RNA detection and quantification are pivotal for diagnosis and monitoring of response to newly approved treatment. We evaluate the performance of three HDV RNA detection and quantification assays.

**Methods:**

Hepatitis Delta RT-PCR system kit, EurobioPlex HDV assay, and RoboGene HDV RNA Quantification kit 2.0 were used for testing 151 HBsAg-positive samples, 90 HDV-RNA negative and 61 HDV-RNA positive. We also evaluated serial dilutions of the WHO international standard for HDV, PEI 7657/12. All HDV-RNA positive samples were genotyped using a next-generation sequencing strategy.

**Results:**

Qualitative results indicated a 100% concordance between tests. Quantitative results correlated well, r^2^ = 0.703 (Vircell-vs-Eurobio), r^2^ = 0.833 (Vircell-vs-RoboGene), r^2^ = 0.835 (Robogene-vs-Eurobio). Bias index was 2.083 (Vircell-vs-Eurobio), -1.283 (Vircell-vs-RoboGene), and -3.36 (Robogene-vs-Eurobio). Using the WHO IS, Vircell overestimated the viral load by 0.98 log IU/mL, Eurobio by 1.46 log IU/mL, and RoboGene underestimated it by 0.98 log IU/mL. Fifty-nine samples were successfully genotyped (Genotype 1, n=52; Genotype 5, n=7; Genotype 6, n=1), with similar results for correlation and bias.

**Conclusion:**

This study underscores the necessity of using reliable HDV-RNA detection and quantification assays, as evidenced by the high concordance rates in qualitative detection and the observed variability in quantitative results. These findings highlight the importance of consistent assay use in clinical practice to ensure accurate diagnosis and effective treatment monitoring of HDV infection.

## Introduction

Hepatitis delta virus (HDV), belonging to the Herpeviridae family, is a small, defective RNA virus. Its existence is contingent upon concurrent infection with the hepatitis B virus (HBV), either through coinfection or superinfection (Hughes et al, 2011; Negro and Suk-Fong, 2023). The ramifications of HDV infection can be severe, exacerbating chronic HBV infection and accelerating the progression to liver failure, cirrhosis, or hepatocellular carcinoma, especially in younger patients (Rizzetto, 1989; Hughes et al, 2011; Alvarado-Mora et al., 2013; Miao et al., 2020).

HDV is categorized into eight global genotypes, each with distinct geographical distributions, disease severities, and levels of accuracy in viral load quantification. Genotype 1 is notably widespread in Western countries (Rizzetto, 1989; Hughes et al, 2011; Negro and Suk-Fong, 2023). HDV’s estimated prevalence is 0.8% among the general population and rises to 10–13% among those afflicted with HBV, indicating that around 48 to 60 million individuals are affected worldwide (Miao et al., 2020). Nevertheless, it is widely acknowledged that the true prevalence of HDV remains underestimated.

Historically, the primary treatment for HDV has been pegylated interferon alpha, administered for a minimum of 48 weeks. However, the overall virological response rate remains low (Hughes et al, 2011; Alvarado-Mora et al., 2013). Recently, the European Medicines Agency (EMA) granted approval to an antiviral medication for HDV treatment—Bulevirtide (Hepcludex^®^). Its mode of action involves hindering the entry of HBV and HDV into hepatocytes by interacting with and deactivating the sodium/taurocholate cotransporting polypeptide (NTCP), a bile salt transporter functioning as a receptor. This drug is currently indicated for the treatment of chronic HDV infection in adults with positive HDV-RNA in plasma (or serum) and compensated liver disease. An HDV viral load reduction of 2 logs has been proposed as an endpoint to assess drug efficacy (Yurdaydin et al., 2019). Thus, precise HDV-RNA detection and quantification are pivotal for accurate diagnosis and treatment monitoring (Kang et al, 2020; Urban et al, 2021; European Medicines Agency, 2023). However, currently, there are no FDA-approved assays available for HDV-RNA detection and quantification.

Considering the pivotal role of viral load monitoring in evaluating the efficacy of the newly approved HDV treatment, our study aims to evaluate and compare the performance attributes of three HDV RNA detection and quantification assays—two commercially available (EurobioPlex HDV assay and RoboGene HDV RNA Quantification kit 2.0) and a RUO-Research Use Only- (Hepatitis Delta RT-PCR system kit).

## Materials and methods

### Study population and specimens

In our study, a total of 151 clinical samples were included from patients who tested positive for HBsAg. Out of these, 90 samples were derived from patients who were negative for both anti-HDV antibodies and HDV-RNA (45 from serum and 45 from plasma) assessed using the Hepatitis Delta RT-PCR system kit from Vircell (Spain) Additionally, 61 samples of serum or plasma (33 from serum and 28 from plasma) were collected from patients who tested positive for both anti-HDV antibodies and HDV-RNA using the same assay. Among the latter group, 36 samples originated from the Microbiology Department of the Clinical University Hospital San Cecilio in Granada. These samples represented patients from various countries including Spain (n=25), Senegal (n=5), Ukraine (n=2), Western Sahara (n=1), Romania (n=1), Ivory Coast (n=1), and Equatorial Guinea (n=1). The remaining 25 samples were obtained from an international patient collection (Cerba Research Biorepository, Gent, Belgium), with patients hailing from France (n=15), Cameroon (n=5), Romania (n=4), and Mauritania (n=1). Anti-HDV were tested using Liaison XL Murex Anti-HDV (Diasorin).

### Molecular assays for HDV detection and quantification

Our study involved the comparison of three different assays for the detection and quantification of Hepatitis D Virus (HDV). These assays included the Hepatitis Delta RT-PCR system kit from Vircell (Spain), the EurobioPlex HDV qRT-PCR assay from Eurobio Scientific (France), and the RoboGene HDV RNA Quantification kit 2.0 from Roboscreen Diagnostics (Germany). The latter two assays are labeled as Conformité Europeéne (CE) and *in vitro* diagnostics (IVD) tests, designed for HDV detection and quantification in routine clinical practice.

Before conducting reverse transcription and amplification with all assays, a nucleic acid extraction was performed on 300 μL of samples using the Maelstrom 4810 system (TANBead). The resulting nucleic acids were eluted in a volume of 60 μL. Real-time PCRs were carried out using a CFX-96 real-time thermocycler (Bio-Rad^®^, CA, USA). Each assay was conducted using the same RNA eluate for consistency following the PCR profile indicated in manufacturer´s instructions and maintaining the sample and master mix ratio recommended. Specifically, 5μl of RNA eluate was used for the Hepatitis Delta RT-PCR system kit and the RoboGene HDV RNA Quantification test, while 10μl of eluate was required for the EurobioPlex HDV assay.

Additionally, we evaluated the WHO international standard for HDV, PEI 7657/12, with a concentration of 575,000 IU/ml. Three serial dilutions of this standard were tested in triplicate, with concentrations of 5,750 IU/ml (3.76 log IU/mL), 575 IU/ml (2.76 log IU/mL), and 23 IU/ml (1.36 log IU/mL) respectively

The Vircell assay utilizes real-time PCR for the detection and quantification of HDV-RNA. It targets a specific region of the HDV genome (*HDAg-L* gene) and can be applied to both serum and plasma samples. The LoD of the assay is 23 IU/mL. The EurobioPlex HDV assay features primers and probes designed for the HDV antigen-coding region. Its sensitivity and specificity stand at 97.7% and 93.4% respectively, with a Limit of Detection (LOD) of 1x10^2^ IU/mL (Le Gal et al., 2017; EurobioPlex HDV, 2023). The RoboGene HDV RNA Quantification test employs primers and probes specific to a subsequence of the Hepatitis delta antigen. This kit boasts a 100% specificity, and its sensitivity and LOD vary depending on the real-time PCR instrument and purification kit utilized (Roboscreen Diagnostics, 2020).

The RoboGene test was selected as the gold standard for the evaluation of sensitivity, specificity, positive predictive value (PPV), and negative predictive value (NPV) for several well-founded reasons, such as its widespread clinical acceptance and its validated performance.

### Statistical analysis

All the graphs, calculations, and statistical analyses were performed using GraphPad Prism software version 8.0 (GraphPad Software, San Diego, CA, USA). For qualitative variables, a concordance analysis was carried out, and the results were expressed as a percentage. To explore the correlation between quantitative results, which were transformed into Log IU/mL values, a linear regression analysis was conducted. This analysis aimed to assess the goodness-of-fit, and the correlation coefficient (r^2^) was calculated. Additionally, the Bland-Altman plot was generated to determine the Bias index. By plotting assay results against each other, a regression analysis was performed to compute the correlation coefficient.

### HDV genotyping

For the purpose of genotyping, a sequencing strategy based on near full-length amplicons with overlapping primers was employed (Çelik et al., 2011). Subsequently, these amplicons underwent sequencing using Illumina’s tagmentation-indexing strategy, and the resulting libraries were processed using a NextSeq 1000 system. The assembly process involved the utilization of CLC-Genomics-Workbench software, which employed reference sequences from various genotypes sourced from the Hepatitis Delta Virus Database (https://hdvdb.bio.wzw.tum.de/hdvdb/).

### Ethics approval

The study adhered to the principles outlined in the Declaration of Helsinki and was both designed and conducted accordingly. It received approval from the local Ethics Committee of Hospital Universitario Clínico San Cecilio in Granada. Due to the deidentified nature of the testing conducted, this ethics committee deemed individual patient consent unnecessary for this study.

## Results

### Concordance between tests

#### Qualitative results

Among the 151 samples assessed, qualitative results indicated that 61 samples were positive using the Vircell assay, 61 with the EurobioPlex HDV kit, and 60 with the RoboGene HDV assay (one positive sample by Vircell & Eurobio could not be tested with Robogene, due to the lack of sample to perform PCR testing). Using the RoboGene test as the reference, the overall concordance rates were 100% for Vircell and 100% for EurobioPlex. Sensitivity, specificity, positive predictive value, and negative predictive value, along with their 95% confidence intervals, were 100% (94.1 - 100%), 100% (96.0 - 100%), 100% (94.1 - 100%), 100% (96.0 - 100%) for the EurobioPlex assay, respectively. For the Vircell assay, the values were 100% (94.0 - 100%), 100% (96.0 – 100%), 100% (94.0 –100%), and 100% (96.0 - 100%), respectively. A detailed presentation of these findings can be seen in **Table 1**.

**Table 1:** Analytical performance and agreement between the three kits compared.

RoboGene HDV RNA quantification kit (Roboscreen diagnostics^®^)*								
		Pos	Neg	Concordance	Sensitivity (95% CI)	Specificity (95%CI)	PPV (95% CI)	NPV (95% CI)
**EurobioPlex HDV**	**Pos**	61	–	100%	100% (94.1 - 100%)	100% (96.0 -100%)	100% (94.1 - 100%)	100% (96.0 -100%)
	**Neg**	–	90					
**Vircell Delta RNA**	**Pos**	60	–	100%	100% (94.0 - 100%)	100% (96.0 – 100%)	100% (94.0 – 100%)	100% (96.0 -100%)
	**Neg**	–	90					

* one positive sample by Vircell & Eurobio could not be tested with Robogene.

#### Quantitative *results*

**Figure 1:**
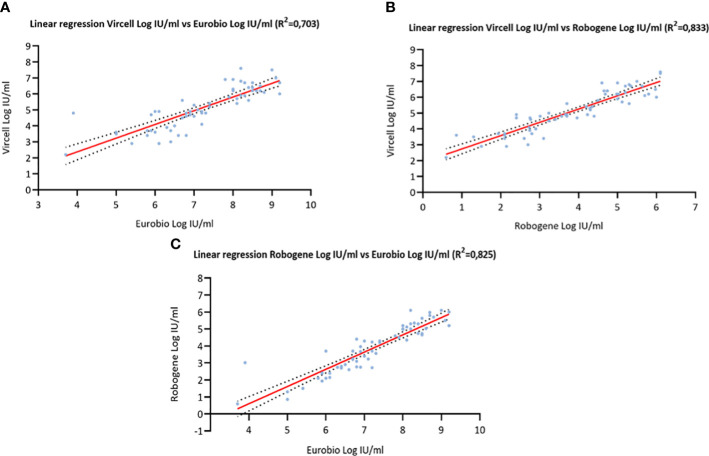
Log IU/ml-transformed quantitative viral load agreement determined by: **(A)** Hepatitis Delta RT-PCR system kit (Vircell, Spain) and EurobioPlex HDV assay (Eurobio Scientific, France); **(B)** Hepatitis Delta RT-PCR system kit (Vircell, Spain) and RoboGene HDV RNA Quantification kit (Roboscreen Diagnostics, Germany). **(C)** EurobioPlex HDV assay (Eurobio Scientific, France) vs RoboGene HDV RNA Quantification kit (Roboscreen Diagnostics, Germany).

**Figure 2:**
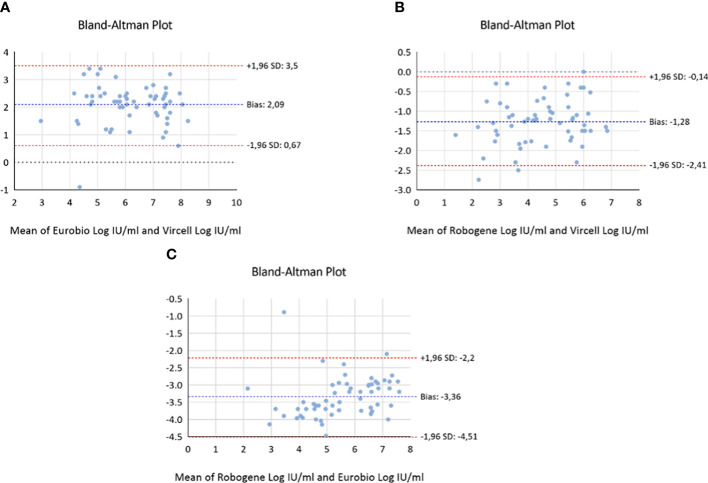
Bland-Altman plot representing the differences found between: **(A)** Hepatitis Delta RT-PCR system kit (Vircell, Spain) and EurobioPlex HDV assay (Eurobio Scientific, France); **(B)** Hepatitis Delta RT-PCR system kit (Vircell, Spain) and RoboGene HDV RNA Quantification kit (Roboscreen Diagnostics, Germany). **(C)** EurobioPlex HDV assay (Eurobio Scientific, France) vs RoboGene HDV RNA Quantification kit (Roboscreen Diagnostics, Germany).

A goodness-of-fit analysis was employed to determine the correlation coefficients (r^2^) between the three tests (**Figure 1**). The calculated coefficients were as follows: r^2^ = 0.703 for Vircell versus Eurobio, r^2^ = 0.833 for Vircell versus RoboGene, and r^2^ = 0.835 for Robogene versus Eurobio. The bias index, determined through a Bland-Altman analysis, produced results of 2.083 for Vircell versus Eurobio, -1.283 for Vircell versus RoboGene, and -3.36 for Robogene versus Eurobio (**Figure 2**).Assay Comparison using WHO International Standard

**Table 2** presents results obtained for the three serial dilutions of the WHO international standard (IS) tested concurrently by the three kits. For an IS concentration of 5750 IU/mL (3.76 log IU/mL), the differences in log IU/mL quantification were -0.82 for Vircell, 1.26 for RoboGene, and 0.83 for Eurobio. At an IS concentration of 575 IU/mL (2.76 log IU/mL), the differences were -1.07 for Vircell, 1.57 for RoboGene, and 0.11 for Eurobio. Lastly, for an IS concentration of 23 IU/mL (1.36 log IU/mL), the differences were -0.98 for Vircell, 0.89 for RoboGene, and -1.06 for Eurobio. In summary, the Vircell kit overestimated the viral load by 0.98 log IU/mL, the Eurobio assay by 1.46 log IU/mL, and the RoboGene kit underestimated it by 0.98 log IU/mL.

**Table 2:** Results obtained by testing serial dilutions of the international standard in parallel by the three kits compared.

		IS Concentration		
		5750 IU/mL (3,76 log IU/mL)	575 IU/mL (2,76 log IU/mL)	23 IU/mL (1,36 log IU/mL)
**Vircell Assay**	**Quantification log IU/mL**	4,58	3,83	2,34
	**Difference with IS (log IU/mL)**	-0,82	-1,07	-0,98
**RoboGene Assay**	**Quantification log IU/mL**	2,5	1,19	0,47
	**Difference with IS (log IU/mL)**	1,26	1,57	0,89
**Eurobio Assay**	**Quantification log IU/mL**	2,93	2,65	2,82
	**Difference with IS (log IU/mL)**	0,83	0,11	-1,06
**RoboGene-Vircell**	**Difference log IU/mL**	-2,08	-2,63	-1,87
**Eurobio-Vircell**		-1,64	-1,18	0,48
**Eurobio-RoboGene**		0,44	1,46	2,36

### Genotyping analysis

Among the 59 samples subjected to full HDV genome sequencing, 51 were successfully classified as Genotype 1, 7 as Genotype 5, and 1 as Genotype 6. Samples classified as Genotype 1 came from Spain (n=20), France (n=4), Cameroon (n=4), Romania (n=2), Ukraine (n=2), Senegal (n=1), Mauritania (n=1), Equatorial Guinea (n=1) and Western Sahara (n=1). Genotype 5 samples originated from patients in France, Senegal, and Ivory Coast, while the Genotype 6 sample was from a Cameroonian patient. For eight samples the genotype could not be ascribed, 2 of them being excluded due to insufficient eluted volume, an additional two samples due to low RNA quantification, and 4 samples due to low quality sequencing. Correlation coefficients for Genotype 1 patients were r^2^ = 0.686 for Vircell versus Eurobio, r^2^ = 0.843 for Vircell versus RoboGene, and r^2^ = 0.799 for Robogene versus Eurobio. Bias index values were 2.1 for Vircell versus Eurobio, -1.31 for Vircell versus RoboGene, and -3.4 for Robogene versus Eurobio. For Genotype 5 patients, correlation coefficients were r^2^ = 0.947 for Vircell versus Eurobio, r^2^ = 0.973 for Vircell versus RoboGene, and r^2^ = 0.991 for Robogene versus Eurobio. Bias index values were 2.21 for Vircell versus Eurobio, -0.79 for Vircell versus RoboGene, and -3 for Robogene versus Eurobio.

## Discussion

HDV exacerbates liver disorders, precipitating severe outcomes like liver failure, cirrhosis, and hepatocellular carcinoma (Da et al, 2019). The limited success of conventional therapies accentuates the urgency for effective options, exemplified by Bulevirtide, heralding a novel era for these challenging patients (Urban et al, 2021). This accentuates the demand for accurate, standardized, and sensitive HDV-RNA assays (Association for the Study of the Liver et al., 2023). Our study meticulously scrutinized three HDV RNA detection assays (Vircell, EurobioPlex, and RoboGene), revealing considerable concordance rates that consistently discerned HDV-positive and HDV-negative samples. Nevertheless, quantitative assessment unveiled noteworthy discrepancies among the assays, reinforcing the imperative of monitoring HDV RNA levels with the same assay and laboratory for treatment monitoring to mitigate inter-laboratory and inter-assay variability.

In our investigation, both the EurobioPlex and Vircell assays achieved high sensitivity and specificity, when compared to the RoboGene assay as the benchmark. The robustness demonstrated by the three assays in identifying chronic HDV infection is remarkable. However, it is important to note that our study did not involve the examination of longitudinal samples from the same patient. Recent research has unveiled a decline in HDV viral load over time in a substantial subset of patients, especially those with cirrhosis (Palom et al., 2021), and is often accompanied by reduced aminotransferase levels, which emphasizes the significance of re-testing serum HDV RNA.

Quantitative analysis provided insights into the correlation and bias between the different assays. Correlation coefficients with values ranging from 0.703 to 0.835, revealed that all the three tests can be considered as highly correlated. Although Bland-Altman plots could reinforce these findings, with in theory acceptable bias indices across the assays, and indicating that while each assay may slightly differ in quantification, they generally yield comparable results, we believe, as others (Association for the Study of the Liver et al., 2023), that for clinical use the observed differences between tests do not warrant interchangeability to monitor the antiviral treatment of chronic HDV. The reduction of HDV replication stands as a pivotal objective in treating HDV infection. Hence, continuous monitoring of viral load throughout treatment utilizing rigorously standardized and validated real-time molecular assays is imperative (Le Gal et al., 2016). To circumvent inter-laboratory discrepancies and mitigate inter-assay variability (Le Gal et al., 2016; Yurdaydin et al., 2019), ensuring accurate and consistent measurements of HDV RNA viral load becomes paramount.

In order to ascertain whether the apparent bias detected during the analysis of clinical samples could be ascribed to the RNA extraction procedure or the specific real-time PCR platform employed (BIO-RAD CFX), we conducted a comparative assessment utilizing the WHO International Standard. Our investigations validated our initial findings, as all three tests consistently exhibited variations (either overestimating or underestimating) across all tested dilutions.

Until there is harmonization across the different assays, quantitative HDV RNA monitoring in sequential serum samples should be performed in the same laboratory and with the same assay to avoid inter-laboratory and interassay variability. This recommendation is crucial for several reasons: a) Inter-Laboratory Variability: Even when the same assay is used, different laboratories may have slight variations in their protocols, equipment calibration, and technician expertise, which can introduce variability in the results. This can lead to inconsistencies in the quantification of HDV RNA levels, potentially affecting clinical decision-making and patient management; b) Inter-Assay Variability: When different assays are used within the same laboratory, there is a risk of obtaining non-comparable results due to differences in assay sensitivity, specificity, and dynamic range. Different assays may target different regions of the HDV genome, have varying amplification efficiencies, and use different detection technologies, all of which can contribute to significant differences in the quantitative results. This variability can complicate the interpretation of viral load trends over time, leading to potential misclassification of the patient’s response to therapy or disease progression; and c) Clinical Implications: Inconsistent HDV RNA measurements can have direct clinical implications, such as incorrect assessment of treatment efficacy, inappropriate changes in therapy, and misinterpretation of virological response or relapse. Reliable and consistent monitoring of HDV RNA levels is essential for making informed clinical decisions, adjusting treatment regimens, and predicting patient outcomes. By performing quantitative HDV RNA monitoring in the same laboratory and with the same assay, we can minimize these sources of variability, ensuring more accurate and reliable results. This approach will enhance the comparability of serial measurements, improve the quality of patient care, and contribute to better clinical outcomes.

In addition, genotyping further enriched the study’s findings. The considerable genetic diversity observed across distinct HDV genotypes and certain sub-genotypes has been demonstrated to contribute to the underestimation of viral load by numerous commercially accessible assays. This effect is particularly notable in instances involving African sub-genotype 1 and African genotypes 5–8 (Brichler et al., 2013). Regrettably, the prevalence of HDV genotypes within our study population was primarily confined to genotype 1. As anticipated, the correlation coefficients and bias indices for this genotype closely mirrored those of the broader global study. In the instances where we could assess a limited number of genotype 5 cases, the correlation was highly satisfactory; however, there remained an observable bias between the tests.

As already discussed, it is important to acknowledge the limitations of our study. The sample size and population diversity, while representative of certain regions, might not fully capture global HDV diversity. Additionally, the assays’ performance might be influenced by various factors like operator experience, laboratory conditions, method of extraction of RNA and platform used for running the PCR.

In conclusion, our study underscores the necessity of using reliable HDV-RNA detection and quantification assays, as evidenced by the high concordance rates in qualitative detection and the observed variability in quantitative results. These findings highlight the importance of consistent assay use in clinical practice to ensure accurate diagnosis and effective treatment monitoring of HDV infection.

Efforts should be directed toward developing standardized HDV detection assays that consider genotypic diversity and global distribution. Collaborative studies involving larger and more diverse patient cohorts could further validate the findings and guide assay selection for specific genotypes and regions.

## Data availability statement

The original contributions presented in the study are publicly available. This data can be found here: 10.6084/m9.figshare.26068306.

## Ethics statement

The studies involving humans were approved by Ethics Committee of Hospital Universitario Clínico San Cecilio in Granada. The studies were conducted in accordance with the local legislation and institutional requirements. Written informed consent for participation was not required from the participants or the participants’ legal guardians/next of kin because due to the deidentified nature of the testing conducted, individual patient consent was not deemed necessary for this study.

## Author contributions

MI-L: Investigation, Writing – original draft. LC-B: Formal analysis, Writing – review & editing. AS: Conceptualization, Writing – review & editing. MH-F: Writing – review & editing. RC: Writing – review & editing. EL: Writing – review & editing. AF: Writing – review & editing. SP: Writing – review & editing. MC: Writing – review & editing. AD: Writing – review & editing. CF: Writing – review & editing. AI: Writing – review & editing. VO: Writing – review & editing. AG-B: Writing – review & editing. AA: Writing – review & editing. MP: Writing – review & editing. FG: Conceptualization, Supervision, Writing – review & editing.
